# Accelerometer-Determined Physical Activity and Its Comparison with the International Physical Activity Questionnaire in a Sample of Nigerian Adults

**DOI:** 10.1371/journal.pone.0087233

**Published:** 2014-01-28

**Authors:** Adewale L. Oyeyemi, Maimuna Umar, Friday Oguche, Salamatu U. Aliyu, Adetoyeje Y. Oyeyemi

**Affiliations:** 1 Department of Physiotherapy, College of Medical Sciences, University of Maiduguri, Maiduguri, Nigeria; 2 Department of Physiotherapy, University of Maiduguri Teaching Hospital, Maiduguri, Nigeria; 3 Department of Physiotherapy, Jos University Teaching Hospital, Jos, Nigeria; Universidad Europea de Madrid, Spain

## Abstract

**Introduction:**

Accurate assessment of physical activity to identify current levels and changes within the population is dependent on the precision of the measurement tools. The aim of this study was to compare components of physical activity measured with an adapted version of the International Physical Activity Questionnaire (Hausa IPAQ-SF) and the accelerometer in a sample of Nigeria adults.

**Methods:**

One hundred and forty-four participants (Mean age = 32.6±9.9 years, 40.3% women) in a cross-sectional study wore an accelerometer for seven consecutive days and completed the Hausa IPAQ-SF questionnaire on the eighth day. Total physical activity, time spent in moderate-to-vigorous activity (MVPA) and sedentary time assessed by Hausa IPAQ-SF and accelerometer were compared. The absolute and criterion- related validity of the Hausa IPAQ-SF was assessed by Bland-Altman analysis and Spearman Correlation Coefficients, respectively. Specificity and sensitivity were calculated to classify individuals according to the global standard guideline for sufficient physical activity.

**Results:**

Compared with the accelerometer, higher time in MVPA and total physical activity were reported on the Hausa IPAQ-SF (p<0.001), while low to moderate correlations (Rs = 0.03–0.38) were found between the two methods. The 95% limits of agreement were wide between methods for total physical activity (−23019 to 20375 METmin.d^−1^) and sedentary time (−510 to 150 min.d^−1^). The sensitivity (76.2%) of Hausa IPAQ-SF to identify insufficiently active people was good, but its specificity (33.3%) to correctly classify sufficiently active people was low.

**Conclusions:**

The Hausa IPAQ-SF overestimated components of physical activity among Nigerian adults, and demonstrated poor to moderate evidence of absolute and criterion validity. Further evaluation of IPAQ and other self-report physical activity instruments in other Africa populations could enhance accurate evaluation of physical activity data in the region countries.

## Introduction

Physical activity levels are often monitored to assess health behaviours and their associations with health status, including mortality and morbidity rates in the population [1]–[3]. Accurate assessment of physical activity is required for effective intervention and implementation of non-communicable chronic diseases' (NCDs) prevention programmes [4], but measurement accuracy is dependent on the precision of physical activity tools [5], [6]. Presently, numerous objective and subjective self-report instruments are available for assessing physical activity in the population, and previous studies have observed some differences in physical activity estimates obtained using both measurement types [5], [7]–[11].

Objective measures, such as pedometers, which count steps, and accelerometers, which measure movement intensity have been used to assess physical activity levels in population studies [2]–[14]. The actigraph accelerometer provides objective and valid measure of time spent in sedentary, light, moderate and vigorous- intensities physical activity [15]–[18], and is a good reference method for assessing the validity of physical activity questionnaires [8], [9], [19]. However, the feasibility of using large quantities of accelerometers for population based physical activity assessment in low income countries could be hampered by limited financial resources and expertise [19], [20]. Because of their low cost and ease of use, self-report measures of physical activity could be most feasible for evaluating population's physical activity levels in low income countries of Africa, but such measures must be valid and culturally sensitive to generate meaningful and comparable data.

The International Physical Activity Questionnaire (IPAQ) was developed by the working group of the World Health Organization (WHO) and the Center for Disease Control and Prevention (CDC) in the United States as a self-report measure for standardizing the assessment of the prevalence of physical activity and for assessing comparable population levels of physical activity in different countries and cultures around the world [19]. In this context, the IPAQ has been used extensively for physical activity research in many countries [19], [21]–[24]. However, most of the studies that have validated the IPAQ by comparing its outcomes with those of accelerometers were conducted in Western developed countries [8]–[11], [25], [26]. In the African region, the validity of IPAQ has only been tested in South Africa as part of the initial development process of the questionnaire [19], and quite recently in Nigeria where a cultural adaptation study of the IPAQ-Short Form (SF) was conducted [27].

The Nigerian IPAQ- Short Form (Hausa IPAQ-SF) [27] showed acceptable test-retest reliability similar to the findings in some studies [28]–[31], but findings on its construct validity were inconsistent with those reported in other studies [26], [28], [32]. Construct validity of the adapted Hausa IPAQ-SF in Nigeria was assessed by comparing its items with rate pressure product (product of resting heart rate and systolic blood pressure) and body mass index, and not with objectively measured physical activity as done in most other studies [8]–[11], [26], [28]–[32]. Further evaluation of the validity of the adapted Hausa IPAQ-SF in Nigeria using objective criterion tools like accelerometers is important for determining the relevance of IPAQ as a useful tool for population surveillance of physical activity in the Africa region. Because Nigeria is the most populous country in Africa with culture and languages similar to other West African countries, it is a good choice to evaluate the IPAQ for psychometric relevance in this country. Understanding and evaluating the magnitude of the discrepancies or agreement between self-report and objective physical activity measures are particularly important for accurate analysis of physical activity data in developing Africa countries where the prevalence of inactivity related NCDs is on the rise [1]–[4]. The aim of this study was to compare components of physical activity measured with an adapted version of the Hausa IPAQ-SF and the accelerometer (absolute and criterion-related validity) in a sample of Nigeria adults. As a secondary objective, the specificity and sensitivity of the Hausa IPAQ-SF to accurately classify Nigerian adults according to the global guideline for sufficient physical activity were also investigated.

## Methods

This was an observational cross-sectional designed study. A sample of 144 adults (58 women and 86 men) out of the 193 that were purposively recruited from various residential homes in Maiduguri city provided valid data and participated in the study (response = 74.6%). Maiduguri is the largest and capital city of Borno State in North-Eastern Nigeria. The city has an estimated population of 749,123 people and attracts immigrants from neighbouring countries of Cameroon, Niger and Tchad [33], [34]. The diverse residents of Maiduguri predominantly use the Hausa language as the common means of communication and for commercial activities [34]. Hausa is a widely spoken language in West-Africa with over 50 million native speakers and 15 million non-native speakers in Northern Nigeria, the Republic of Niger, Northern Cameroon and Ghana [35]. Participants were eligible for this study if they were willing to wear Actigraph accelerometer for at least seven consecutive days and were willing to complete surveys in either English or Hausa language. Additional eligibility criteria included being adults (18–65 years) and not having any disability that prevented independent walking. All participants were fully informed of the study protocol and provided written informed consent. The study protocol was approved by the Research and Ethic Committee of the University of Maiduguri Teaching Hospital, Maiduguri, Nigeria.

### Procedure

All participants in the study were visited at home, eight days apart and were requested to wear an Actigraph accelerometer and complete the Hausa version of IPAQ-SF. Prior to the first visit; the accelerometer was initialized and set to record physical activity in 60-second epoch [10], [36]. The participants were instructed to wear the accelerometer attached to an elasticized belt around the waist, positioned just above the right hip, from when they woke up in the morning until bed time at night for 7 consecutive days. This measurement period was to ensure at least 10 hours of wear time per day. The participants were asked to remove the accelerometer any time they were to perform activities that involve the use of water such as bathing or swimming, and when going to bed. On the 8^th^ day, the participants were visited for the second time to collect the accelerometer, and demographic questionnaire including the Hausa IPAQ-SF was administered. The participants' height and weight were also measured using standardized instruments. Body mass index (BMI) was calculated as body weight divided by the square of height (kg m^−2^), and subjects were consequently placed in normal weight (BMI: 18.5–25 kg m^−2^) or overweight/obese (BMI: >25 kg m^−2^) groups [37].

### Measures

#### Actigraph Accelerometer

Objective measurement of physical activity was provided with Actigraph accelerometer (Manufacture Technology Incorporated (MTI), Actigraph LLC, Pensacola, FL, Model 7164).The Actigraph was originally called the Computer Science Applications (CSA) accelerometer but the CSA changed names to the MTI after the company was purchased by Manufacturing Technologies Inc. The technical specifications and performance characteristics of the Actigraph accelerometer have been described elsewhere [36], [38]. Actigraph data were deemed complete if the participants had accelerometer counts for at least 10 hours per day for at least 5 days, including at least 1 weekend day [9], [10], [19], [39]. At least 30 minutes of continuous zero counts were deemed as acceleremoter non-wearing periods. The accelerometer captures minute-by-minutes activity counts that were collapsed into minutes spent in sedentary, moderate and vigorous intensity activity. Based on cut-points derived from previous studies [19], [35], a minute of accelerometer data were coded as either sedentary or moderate, respectively, if it contained less than 100 activity counts/minute or were between 1952–5724 activity counts/minute, while it was coded as vigorous if the activity counts were greater than 5724/minute. Average intensity was calculated taking the total counts divided by the recorded time and this was considered as a weighted measure of total physical activity [9]. The data were scored and interpreted using the “MeterPlus Version 4.2 software from Santech, Inc. (www.meterplussoftware.com). The total minutes spent in objective moderate-to-vigorous physical activity (MVPA) was calculated by summing minutes per week of moderate- and vigorous-intensity activity. According to the new global standard, meeting recommendations on physical activity for health was defıned as engaging in at least 150 minutes of moderate-intensity activity per week, 75 minutes of vigorous-intensity activity per week, or an equivalent combination of moderate- and vigorous-intensity activity [40], [41]. Actigraph accelerometer provides valid estimate of physical activity [15], [17], and correlates highly with heart rate and with other movement and energy-expenditure estimates [16].

#### International physical activity questionnaire

The Hausa version of the International Physical Activity Questionnaire- Short Form (Hausa IPAQ-SF) which included 7 items was used to assess participants' self-reported physical activity. A detailed description of the adaptation of the Hausa IPAQ-SF has been published [27]. Briefly, the original IPAQ-SF in English language was translated into the Hausa language by two independent translators, synthesized by a panel of six experts that included the translators, back translated by another two translators, and subsequently subjected to expert committee review and pre-testing. The final product (Hausa IPAQ-SF) available in both Hausa and English language assesses vigorous- and moderate- intensity activities, and walking in bouts of at least 10 minutes, in terms of frequency (days/wk) and duration (min/day) in the previous seven days (the period for which the accelerometer was also worn). The Hausa IPAQ-SF also assesses the time spent in sitting (sedentary time) on a weekday during the previous week. To calculate total physical activity per day, vigorous-intensity, moderate- intensity and walking activity were multiplied by their estimated intensity in METs and summed to gain an overall estimate of total physical activity (www.ipaq.ki.se). One MET represents the energy expended while sitting quietly at rest and is equivalent to 3.5 ml/kg/min of VO_2_ [41]. The MET intensities used to score IPAQ were vigorous (8METs), moderate (4METs) and walking (3.3 METs) (www.ipaq.ki.se). MVPA (excluding walking) was computed by summing minutes of time per day of moderate- and vigorous-intensity activity. The test retest reliability (ICC = 0.33–0.73) and concurrent validity (ρ = 0.78–0.92) of the Hausa IPAQ-SF among Nigerian adults Nigeria are good and acceptable [27].

#### Socio-demographic Characteristics

Socio-demographic data included questions about age in years, sex, ethnic group, marital status, educational level and occupational status. The participants were categorized into 18–29 years, 30–45 years and >45 years age groups, and were classified as single or married according to their marital status. Participants were also classified according to their education into more than secondary school education, secondary school education, and less than secondary school education. For occupation, participants were grouped as white collar (government and private company employees), blue collar (self-employed, artisans, traders etc) or unemployed (homemaker, student, or retired).

### Statistical analysis

Descriptive statistics of mean and percentage were used to describe the sociodemographic characteristics of participants. The outcomes from Hausa IPAQ-SF and accelerometer were described by mean, standard deviation (SD) and interquartile ranges (IQR) in total and by gender, age, BMI and education. The differences between the Hausa IPAQ-SF and accelerometer scores were tested with a paired non parametric Wilcoxon test. Comparisons between the two instruments were done using Spearman's rank correlation coefficients (Rs) (criterion validity). The Bland and Altman method was used to provide an indication of the systematic and random error and the heteroscedasticity of the Hausa IPAQ-SF and accelerometer as measures of physical activity, and 95% limits of agreement were used for describing the total error between the two methods (absolute validity). Variables used for the Bland and Altman analysis were daily time spent in moderate-to-vigorous activity (MVPA), sitting and total physical activity according to Hausa IPAQ-SF and accelerometer. In addition, concordance between number of individuals meeting or not meeting the current public health physical activity guidelines [40], [41], as determined by the two different methods was assessed with the Chi-square test. Sensitivity (ability of the Hausa IPAQ-SF to identify insufficiently physically active individuals) and specificity (ability of the Hausa IPAQ-SF to identify sufficiently active individual) were determined. All statistical analyses were performed using Statistical Package for the Social Science (SPSS), version 15.0 for windows (SPSS Inc., Chicago, Illinois, USA) and the level of significance was set at p<0.05.

## Results

The sociodemographic characteristics of the study sample (N = 144) are reported in table 1. The participants comprised 40.3% women and 59.7% men, with a mean age of 32.5±9.9 years and a mean BMI of 23.8±3.9 kg/m^2^. About one-third of the participants were overweight or obese (33.3%, n = 41) and more than half of them had at least a secondary school education (53.5%, n = 77) and were married (57.6%, n = 83).

**Table 1:** Descriptive characteristics of the sample.

Variables	Subcategory	Total Sample	Men	Women
		(N = 144)	(n = 86, 59.7%)	(n = 58, 40.3%)
Age (years)	Mean (±SD)	32.6±9.9	32.9±9.4	32.1±10.6
Age group (n, %)*	18–29	75 (52.1)	40 (46.5)	35 (60.3)
	30–45	48 (33.3)	36 (41.9)	12 (20.7)
	>45	21 (14.6)	10 (11.6)	11 (19.0)
Marital Status (n, %)	Single	61 (42.4)	41 (47.7)	20 (34.5)
	Married	83 (57.6)	45 (52.3)	38 (65.5)
BMI (Kg/m^2^)	Mean (±SD)	23.8±3.7	23.3±3.6	24.5±3.8
BMI Category (n, %)*	Normal Weight	82 (66.7)	58 (75.3)	24 (52.2)
	Overweight/obese	41 (33.3)	19 (24.7)	22 (47.8)
Ethnicity (n, %)	Hausa/Fulani	29 (20.1)	15 (17.4)	14 (24.1)
	Igbo	12 (8.3)	6 (7.0)	6 (10.3)
	Yoruba	8 (5.6)	6 (7.0)	2 (3.4)
	Kanuri/Shuwa Arab	39 (27.1)	26 (30.2)	13 (22.4)
	Others	56 (38.9)	33 (38.4)	23 (39.7)
Educational level (n, %)	<Secondary School	28 (19.4)	11 (12.8)	17 (29.3)
	Secondary School	77 (53.5)	49 (57.0)	28 (48.3)
	>Secondary School	39 (27.1)	26 (30.2)	13 (22.4)
Occupational status (n, %)*	Unemployed	59 (41.0)	19 (22.1)	40 (69.0)
	Blue Collar	63 (43.8)	51 (59.3)	12 (20.7)
	White Collar	22 (15.3)	16 (18.6)	6 (10.3)

*- Significant difference exists between men and women at p<0.05.

SD- Standard Deviation.

BMI- Body Mass Index.

Table 2 shows the descriptive data for the Hausa IPAQ-SF and the accelerometer stratified by gender, age, BMI and education. Across all stratified variables, the Hausa IPAQ-SF when compared to accelerometer showed significantly higher minutes per day in moderate, vigorous, MVPA and total physical activity (p<0.001). Additionally, standard deviations around the means were substantially greater for the Hausa IPAQ-SF, indicating greater variance in self-reported activity levels. Men, younger adults (18–29 years), and those with secondary school education spent more time in total physical activity as reported on the Hausa IPAQ-SF, and this was consistent with patterns on the accelerometer. Minutes of sedentary time per day reported on the Hausa IPAQ-SF was significantly lower compared with the sedentary time recorded on the accelerometer, and this was consistent across all stratified demographic variables (p<0.001).

**Table 2:** Descriptive physical activity data from IPAQ and Actigraph, by sex, age, BMI and education; Mean (SD) and Interquartile Ranges.

	Total PA*		Vigorous PA*		Moderate PA*		MVPA*		Sitting*	
Variables	IPAQ MET min.d^−1^	Actigraph† counts.min^−1^	IPAQ min.d^−1^	Actigraph† min.d^−1^	IPAQ min.d^−1^	Actigraph† min.d^−1^	IPAQ min.d^−1^	Actigraph† min.d^−1^	IPAQ min.d^−1^	Actigraph† min.d^−1^
**All** ‡	6432 (4901)	4722 (9381)	32 (26)	5 (38)	98 (54) 60;	44 (25)	119 (66)	48 (49)	230 (136)	409 (102)
*IQR*	3024; 8415	2947; 4575	15; 45	0; 1	120	22; 62	60; 180	22; 64	120; 360	346; 468)
**Sex**					94					
Men‡	7032 (5205)	5774 (12025)	29 (20)	7 (49)	(55) 60;	55 (24)	115 (64)	62 (59)	210 (133)	405 (109)
*IQR*	3706; 9786	3303; 5004	15; 40	0; 2	120	38; 72	60; 180	40; 73	120; 300	345; 469
Women‡	5441 (4225)	3162 (1020)	37 (35)	1 (3)	104 (51)	27 (15)	124 (69)	28 (16)	260 (137)	415 (91)
*IQR*	2499; 6906	2415; 3771	10; 60	0;1	60; 150	16; 37	70; 180	17; 37	173; 360	346; 455
**Age (years)**					99					
18–29‡	7832 (4626)	5307 (12255)	30 (22)	8 (52)	(52) 0; 120	46 (25)	116 (59)	54 (63)	200 (120)	405 (111)
*IQR*	4079;11774	3030;4587	10; 45	0;2		23; 66	66; 180	23; 69	120; 300	341; 469
30–45‡	6996 (5547)	4299 (5428)	33 (32)	1 (3)	94 (58) 50;	41 (24)	118 (78)	43 (26)	246 (147)	411 (92)
*IQR*	3186; 8364	2783; 4456	15; 14	0;1	180	50; 180	60; 180	22; 59	120; 360	348; 456
>45‡	5642 (4434)	3600 (1171)	32 (19)	1 (2)	101 (50)	40 (26)	126 (61)	41 (27)	290 (143)	417 (88)
*IQR*	2565; 6636	2799; 4537	20; 45	0; 1	60; 120	18; 62	80; 164	60; 180	150; 420	346; 484
**BMI (kgm^−2^)**					96					
<25‡	7032 (5811)	5504 (12361)	30 (19)	7 (50)	(55) 60;	46 (25)	114 (65)	53 (61)	212 (123)	414 (76)
*IQR*	2884;10326	2953; 4503	17; 40	0; 1	120	28; 63	60; 180	28; 66	120; 300	352; 487
>25‡	6246 (3472)	3596 (994)	33 (36)	1 (3)	109 (53)	39 (23)	134 (70)	41 (23)	301 (141)	419 (114)
*IQR*	3510; 8433	2879; 4472	14; 46	0; 1	60; 180	23; 58	75; 181	23; 60	180; 420	347; 464
**Education**					110					
<Secondary‡	6237 (3593)	5208 (6971	24 (17)	1(4)	(49) 60;	40 (26)	120 (60)	41 (29)	266 (129)	374 (71)
*IQR*	3204; 8112	3171; 4773	10; 37	0; 1	180	17; 59	77; 50	18; 60	120; 360	314; 441
Secondary‡	6230 (4386)	5096 (12117)	30 (28)	8 (52)	97 (56) 60;	47 (25)	119 (71)	55 (63)	205 (142)	427 (120)
*IQR*	2537; 8883	2867; 4541	15; 36	0; 1	150	24; 67	60; 180	25; 69	120; 300	347; 505
>Secondary‡	7018 (6681)	3636 (1173)	39 (24)	1 (2)	90 (52) 57;	40 (23)	117 (63)	41 (25)	254 (121)	400 (70)
*IQR*	3075; 7782	3698; 4388)	20; 60	0; 1	120	21; 55	60; 170	21; 56	120; 360	336; 446

* - Significant difference between IPAQ-Actigraph tested for total PA, vigorous, moderate, Moderate-to-Vigorous PA, and sitting, respectively, using paired non parametric Wilcoxon test, *p*<0.001;

† - Cut-off values for sitting, moderate and vigorous were <100, 761–5724, and >5724 counts, respectively;

‡ _ Mean (±Standard deviation); *IQR*_ Interquartile range; BMI_ Body Mass Index; PA_ Physical.

Table 3 shows the Spearman rank order correlations for the time spent in physical activity from the Hausa IPAQ-SF with time spent in similar activities from the accelerometer. Moderate, but significant correlations were observed for MVPA between the Hausa IPAQ-SF and the accelerometer (Rs = 0.15, p = 0.03). Correlations were also statistically significant between total physical activity on the Hausa IPAQ-SF (MET-minutes per day) and the accelerometer total counts per minutes (Rs = 0.38, p<0.01). Similar moderate correlation coefficients were observed by gender, but correlation coefficients were stronger for men than women.

**Table 3:** Spearman Rank Correlation Coefficient (Rs) for Total Physical Activity and Time Spent in Physical Activity from IPAQ and Actigraph (n = 144).

Variables		Spearman correlation coefficients		
IPAQ (min.d-1)	Actigraph (min.d-1)†	All (n = 144)	Women (n = 86)	Men (n = 58)
Vigorous	Vigorous	0.11	0.08	0.19*
Moderate	Moderate	0.04	0.05	0.17
MVPA	MVPA	0.15*	0.14	0.26*
Total PA‡	Total count‡‡	0.38**	0.24*	0.48**
Time spent sitting	Sitting	0.06	0.14	−0.08

† _Cut-off values for sitting, moderate, and vigorous were <100, 1952–5734, and >5724, respectively.

‡ _IPAQ total PA (MET-min.d-1).

‡‡ _Actigraph Total Count (count.min-1).

* _p<0.05;

** _p<0.01.

MVPA: Moderate-to-vigorous physical activity.

The sensitivity of the Hausa IPAQ-SF to capture insufficiently active individuals was 76.2%. Whereas, 33.3% (the specificity) of those meeting the international recommended guidelines as determined by the accelerometer were captured by the Hausa IPAQ-SF (*x* ^2^ = 1.272, p = 0.259). The positive and negative predictive values of the IPAQ-SF were 76.2% and 33.3%, respectively (Table 4).

**Table 4:** Number (%) of participants classified as being sufficiently active according to physical activity guidelines by self-report (Hausa IPAQ-SF) and by accelerometry (Actigraph).

	Meeting PA guideline, Hausa IPAQ-SF		
Meeting PA guideline, accelerometer	No (%)	Yes (%)	Total (%)
No	32 (76.2)	68 (66.7)	100 (69.4)
Yes	10 (23.8)	34 (33.3)	44 (30.6)
Total	42 (100)	102 (100)	144 (100)

PA: Physical activity.

**Figure 1:**
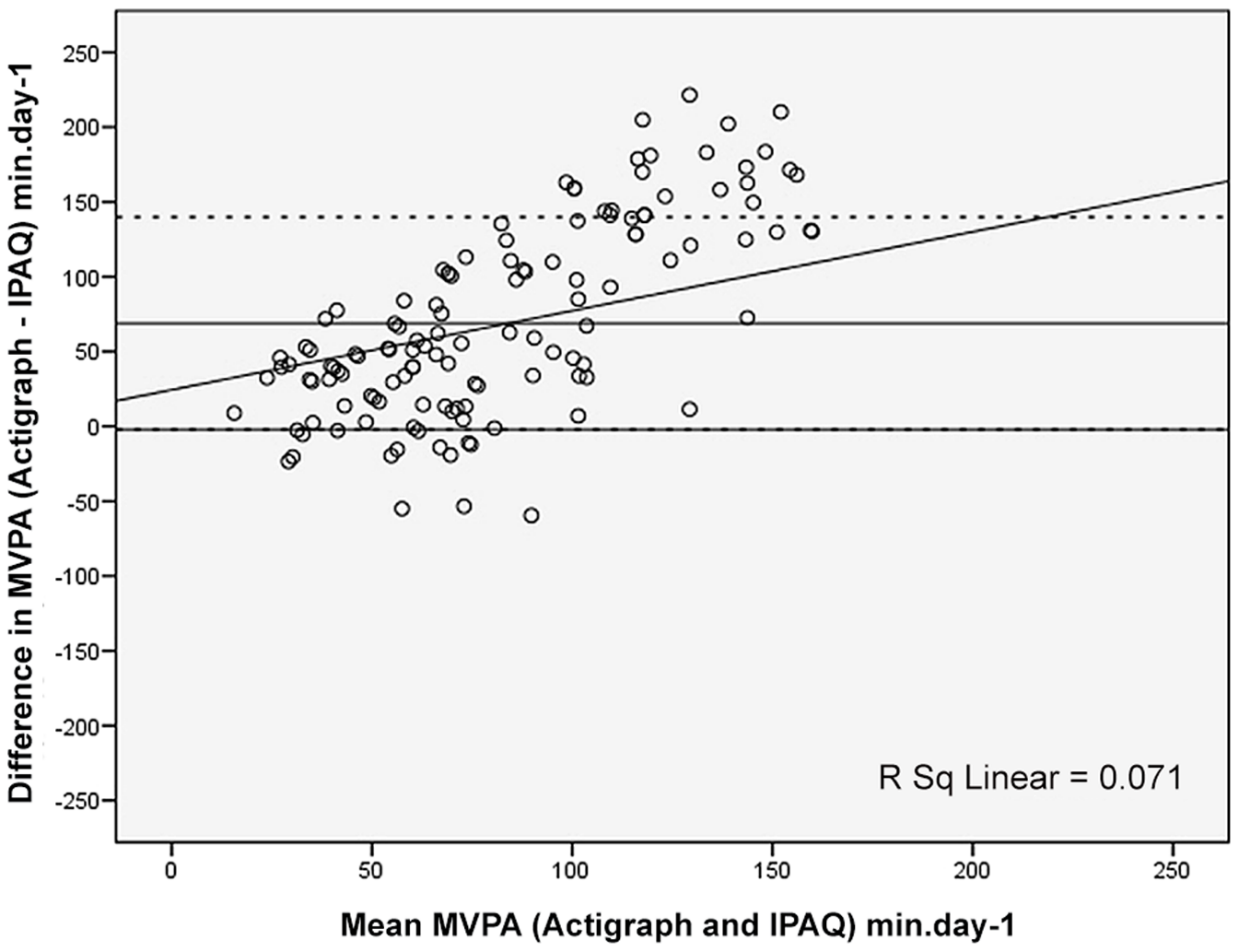
Bland-Altman plot for min.day^−1^ reported in Moderate-to-Vigorous (MVPA) physical activity from the Actigraph and IPAQ. The mean error scores are illustrated by a solid horizontal line and the limits of agreement (±1.96 SD from the mean) are shown as dashed horizontal lines.

**Figure 2:**
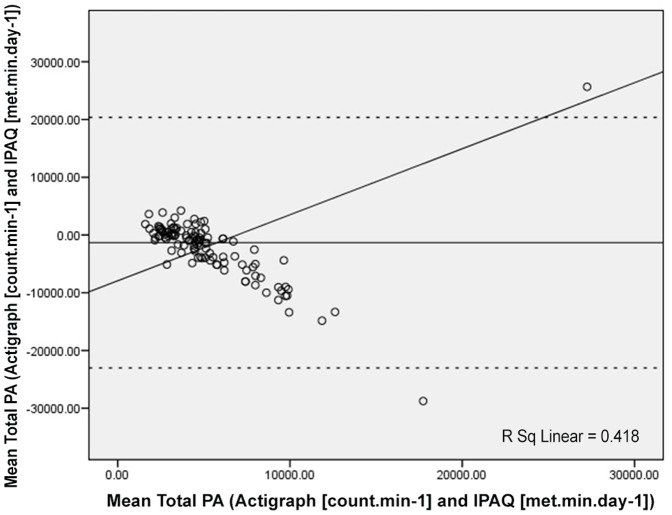
Bland-Altman plot for min.day^−1^ reported in total physical activity (PA) from the Actigraph and IPAQ. The mean error scores are illustrated by a solid horizontal line and the limits of agreement (±1.96 SD from the mean) are shown as dashed horizontal lines.

**Figure 3:**
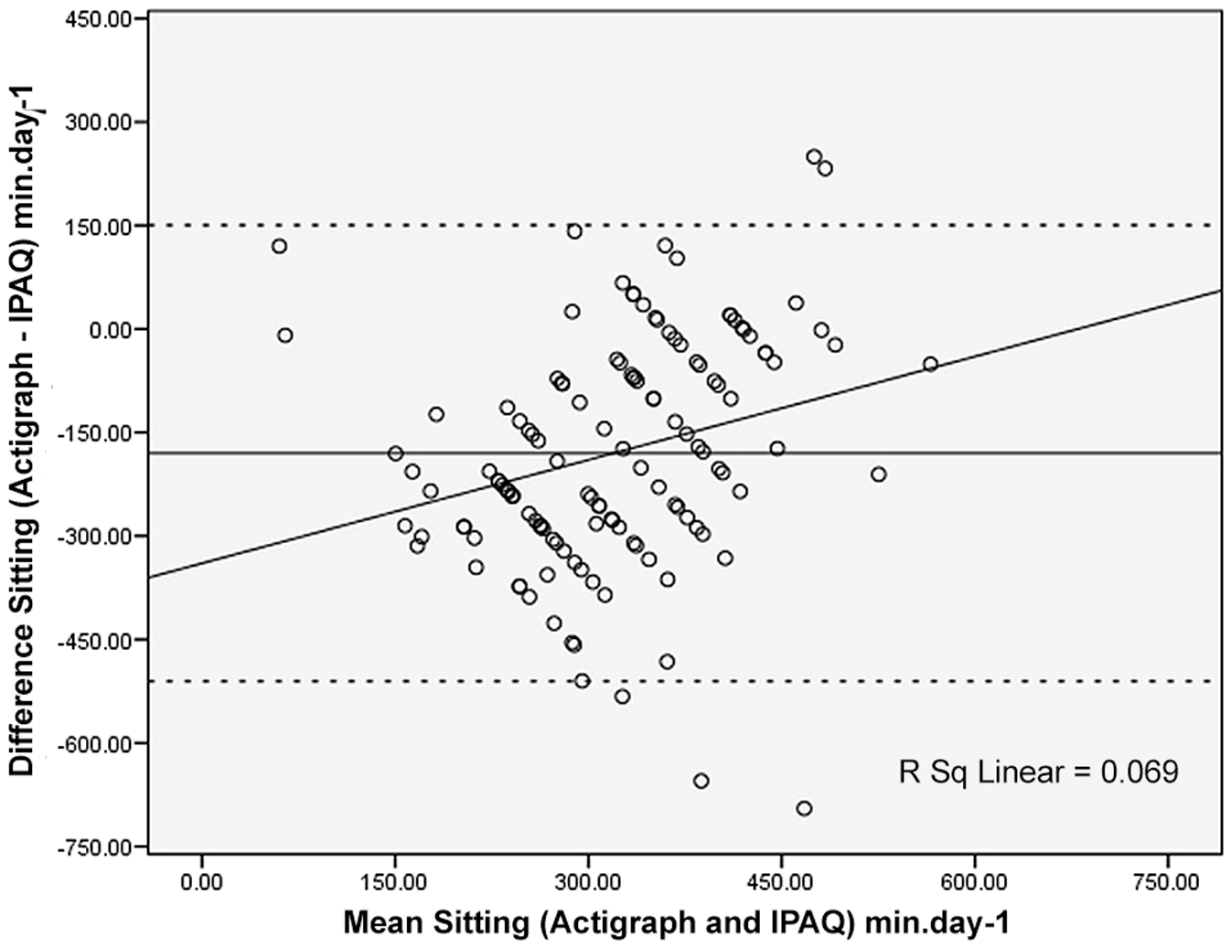
Bland-Altman plot for min.day^−1^ reported for sitting time from the Actigraph and IPAQ. The mean error scores are illustrated by a solid horizontal line and the limits of agreement (±1.96 SD from the mean) are shown as dashed horizontal lines.

Figures 1, 2 and 3 (Bland-Altman plots) illustrate the differences between the Hausa IPAQ-SF and accelerometer minutes per day plotted against the mean of the Hausa IPAQ-SF and accelerometer for MVPA, sitting and total physical activity, respectively.

For MVPA, the mean difference was 69 min.d^−1^ (*p* = 0.10) and the 95% limits of agreement were within reasonable range (−2 to 140 min.d^−1^). Differences (i.e., error) between the Hausa IPAQ-SF and accelerometer scores slightly increased as the minutes per day in MVPA reported on the Hausa IPAQ-SF increased (R^2^ = 0.07) (Fig 1).

In figure 2, the Bland-Altman plot for total physical activity MET-min.d^−1^ from the Hausa IPAQ-SF and the accelerometer (count.min^−1^) showed a mean difference (SD) of 1322 (10848) MET-min.d^−1^ (*p* = 0.08), and the 95% limits of agreement were wide (−23019 to 20375 METmin.d^−1^). Differences (i.e., error) between the Hausa IPAQ-SF and accelerometer scores increased as the total physical activity activities reported in the Hausa IPAQ-SF increased (R^2^ = 0.42).

In figure 3, the Bland-Altman plot for sitting activities from the IPAQ-SF and the Actigraph showed that the mean difference was −180 min.d^−1^ (*p* = 0.13) and the 95% limits of agreement were wide (−510 to 150 min.d^−1^).

## Discussion

This study compared the physical activity scores arising from the Hausa IPAQ-SF and an objective measure (accelerometer) of physical activity in a sample of Nigerian adults. The results showed that compared to components of physical activity measured with accelerometer, significantly more time was reported in moderate-to-vigorous activity (MVPA) and total physical activity, but lower time in sedentary activities on the Hausa IPAQ-SF. There were wide limits of agreement in mean scores between the two methods for total physical activity and sedentary time, suggesting that the absolute validity of the Hausa IPAQ-SF is probably poor to modest.

The Hausa IPAQ-SF produced greater variation in estimates of physical activity than the accelerometer, and the differences in activity minutes reported on the IPAQ as compared with accelerometer increased with higher IPAQ scores. This finding seems to reflect the usual over reporting or failure to correctly recall activity from self-report measure of physical activity [6], [12], [42]. Social desirability bias and coding errors due to possible over inflation of physical activity behaviours have been documented with the IPAQ [42]–[44]. However, it is also possible that the accelerometer, unlike the IPAQ-SF was unable to capture or underestimated the context specific physical activities like bicycling, heavy lifting, manual work and household chores that could have been performed by the participants in the present study. Accelerometers compared to self-report measures of physical activity do not provide details information on the type of activity performed [19].

Despite the wide limits of agreement observed in the Bland-Altman plots, the scores reported for total physical activity from the Hausa IPAQ-SF tend to follow similar demographic patterns as recorded on the accelerometer. Total physical activity reported on both instruments decreased with increasing age, higher BMI, and was lower among women than men. This finding suggests the feasibility of using the Hausa IPAQ-SF to study demographic patterns of overall physical activity levels in Nigeria. However, the pattern of MVPA reported on the Hausa IPAQ-SF was not consistent with that of accelerometer. While women reported more MVPA on IPAQ men had higher scores on accelerometer based MVPA; with similar discrepant patterns recorded for age group, BMI status and education. These demographic discrepancies in MVPA between the two methods could suggest that the Hausa IPAQ-SF may not be optimal for assessing health related physical activity (MVPA) at individual level basis. Previous IPAQ validation studies have also reported limited utility for IPAQ in assessing physical activity at the individual level [8], [10], [11]. The IPAQ-SF was primarily designed for assessing population surveillance of physical activity and not as an evaluation tool for individual based interventions [19].

Correlations between the IPAQ and the accelerometer scores ranged from 0.03 to 0.38, indicating low to moderate relationship between the two instruments. Although only the associations between the Hausa IPAQ-SF and accelerometer scores on total physical activity and MVPA were significant in the present study, the observed correlation coefficients values are similar to those reported in a 12-country validation study of IPAQ [19], and in two Swedish studies [9], [10]. The overall tenuous correlation coefficients found for the components of physical activity in our study is explainable. For long, It has been documented that people generally tend to over-estimate time spent in self-reported high-intensity physical activity but underreport the time spent in light and moderate-intensity activities [6], [45]. On the other hand, accelerometers tend to underscore high intensity physical activities but over-score low intensity physical activities [45], [46]. Moreover, unlike high intensity activities that are more structured, stable over time and much more easily recalled; it is difficult to recall and obtain a good measure of low and moderate- intensity physical activities using self-administered questionnaires [47], [48]. The higher correlations found in the present study for vigorous intensity compared to moderate intensity physical activity seem to confirm these assertions from previous studies. Also consistent with other studies that stratified results by gender [9], [11], we found higher and stronger correlation coefficients for all components of physical activity among men than women. However, the overall low levels of agreements (albeit significant) found between methods call for caution on the suitability of the IPAQ for surveillance of physical activity and for making public health recommendations.

The Hausa IPAQ-SF correctly classified 76% of participants as insufficiently physically active but only captured about a third of those who reported sufficient physical activity according to the current public health guidelines by accelerometer, implying its good sensitivity and low specificity, respectively. It is difficult to speculate on the direction of this finding because only few validation studies on specificity and sensitivity of IPAQ have been conducted and results have been inconsistent [10], [43]. In contrast to our finding, a Swedish study found the IPAQ-SF to provide reasonable specific measure of physical activity but to have limited sensitivity to correctly classify people as insufficiently active [10]. Another study conducted in the United States found the IPAQ to have poor specificity and sensitivity compared to other self-report physical activity measure for predicting attainment of health related physical activity guideline [43]. More studies are needed to clarify the direction and interpretation of findings in this context.

In addition to the Hausa IPAQ-SF being able to correctly classify inactive participants (good sensitivity) in the present study, it also yielded similar patterns of sedentary time as captured by accelerometer across the demographic group (sex, age, BMI and education). For example, with the IPAQ, women reported more sedentary time than men which was confirmed by the accelerometer. Further, with increasing age and BMI more sedentary time was reported on the Hausa IPAQ-SF which was also confirmed by the accelerometer. This finding is worthwhile and interesting because the IPAQ sitting items have been considered an advancement that could be useful as part of physical activity–surveillance methods for assessing sedentary behaviour [49]. Because availability, cost and receptivity to wearing accelerometers for the assessment of physical activity have been identified as an important research issue in the African region [19], [20], an easily available, cost effective and valid self-report questionnaire (such as Hausa IPAQ-SF) that assessed sedentary time in a similar pattern with accelerometer could be deemed a valuable instrument for research and practice in Nigeria.

The strength of this study was the use of an objective measure to evaluate the Hausa IPAQ-SF. The participants were asked to wear the accelerometer for one consecutive week and thereafter answer the Hausa IPAQ-SF survey to cover the one week period during which the accelerometer was worn. One additional strength was the exploration of the sedentary behaviour patterns against age, BMI and gender, the direction and magnitude of which were similar between accelerometry and IPAQ. This study was one of the first to examine the absolute validity of a culturally adapted physical activity measure in the African population. It is particularly valuable to evaluate culturally relevant versions of the IPAQ in sub-Sahara Africa because this can enhance local relevance of evidence and interventions. The present results suggest the feasibility that researchers from other African countries can adapt equivalent versions of IPAQ for the assessment of population based physical activity in the Africa region. Limitations of the study include relative small sample size and the use of non-probability sample. The study finding may have limited generalizability to other samples of Nigerians that have different characteristics from this sample. In addition, the majority of participants have more than secondary school education with potentially higher comprehension and recall ability than may be found in the general population. However, recruitment from diverse neighbourhoods and settings allowed for a sample with reasonable heterogeneity in age, occupational status, and ethnic backgrounds and made it possible to stratify the analyses by sociodemographic characteristics.

In conclusion, low to moderate evidence of absolute and criterion validity was found for the Hausa IPAQ-SF, but the tool overestimated the time spent in physical activity and may incorrectly classify Nigerian adults into sufficiently active category. Our findings have implications on the suitability of using the IPAQ-SF for physical activity surveillance and for making public health recommendations in Nigeria. Further investigations on the validity of IPAQ and other self-report physical activity instruments in other African populations could enhance accurate evaluation of physical activity data needed to implement effective prevention and intervention programmes for chronic non-communicable diseases that are rising in the African region.
